# Greening Blocks: A Conceptual Typology of Practical Design Interventions to Integrate Health and Climate Resilience Co-Benefits

**DOI:** 10.3390/ijerph16214241

**Published:** 2019-11-01

**Authors:** Sara Barron, Sophie Nitoslawski, Kathleen L. Wolf, Angie Woo, Erin Desautels, Stephen R. J. Sheppard

**Affiliations:** 1 School of Ecosystem and Forest Sciences, University of Melbourne, Melbourne, VIC 3121, Australia; 2 Faculty of Forestry, University of British Columbia, Vancouver, BC V6T 1Z4, Canada; snito@mail.ubc.ca (S.N.); stephen.sheppard@ubc.ca (S.R.J.S.); 3 College of the Environment, University of Washington, Seattle, WA 98110, USA; kwolf@uw.edu; 4 Fraser Health Authority, Vancouver, BC V5Z 4H5, Canada; angie.woo@fraserhealth.ca; 5 Sustainability Office, City of Surrey, BC V3T 1V8, Canada; EADesautels@surrey.ca

**Keywords:** urban greening, climate change, public health, urban forest, landscape planning, green design

## Abstract

It is increasingly evident that exposure to green landscape elements benefits human health. Urban green space in cities is also recognized as a crucial adaptation response to changes in climate and its subsequent effects. The exploration of conceptual and practical intersections between human health, green spaces, and climate action is needed. Evidence-based guidance is needed for stakeholders, practitioners, designers, and citizens in order to assess and manage urban green spaces that maximize co-benefits for both human health and climate resilience. This paper proposes interventions that provide strategic green space enhancement at the neighborhood and block scale. We propose eight tangible green space interventions and associated metrics to integrate climate resilience and population health co-benefits into urban green space design and planning: *View from within, Plant entrances, Bring nature nearby, Retain the mature, Generate diversity, Create refuge, Connect experiences, and Optimize green infrastructure.* These interventions represent a hierarchy of functional design concepts that respond to experiential qualities and physical/psychological dimensions of health, and which enhance resilience at a range of social scales from the individual to the neighborhood. The interventions also reveal additional research needs in green space design, particularly in neighborhood-level contexts.

## 1. Introduction

Urban green spaces, understood to be areas comprised of vegetation and other natural elements, play an important role in helping cities transition to more resilient, healthier, and sustainable futures. Green space may include parks, community gardens, greenways and trails, stormwater systems, and tree-lined streets [1]. Urban forests are included in this definition, as trees can serve as both structural and organizing elements within urban green landscapes [2]. Urban forests and green spaces provide a suite of benefits and services to city dwellers towards climate resilience [3,4,5]. Along with other nature-based solutions, meaning the strategies that use nature to some degree to address environmental challenges [6], urban forests and green spaces are increasingly recognized as a necessary intervention in the global dialogue on climate solutions.

Numerous studies also indicate the positive effects of urban green space for human health [7,8]. The “biophilia hypothesis”, for example, suggests that humans experience positive emotional responses from views of nature [9]. Other health benefits may relate to opportunities for increased sense of belonging, social cohesion and interaction in urban green spaces [10,11,12,13,14]. Evidence has also shown that the presence of greenery encourages physical activity [15], though results are mixed [16]. Urban trees specifically promote a similar range of health benefits [17].

Urban green provides broad and diverse social, economic, and ecological services compared to more traditional engineered and grey infrastructure [18]. Ecosystem services such as increased stormwater retention, biodiversity, and energy savings must be balanced with disservices such as allergen production [19]. Few studies combine human health and climate concerns within the context of nature-based solutions, and few municipal plans provide actionable strategies and actions for green space solutions targeting both human health and climate resilience benefits [20]. It is also unclear whether urban foresters and green space managers, health authorities, municipal practitioners, community planners and landscape designers are effectively collaborating to enhance green co-benefits.

In response to this challenge, we propose that decision-support tools and clear and compelling guidance can enable interdisciplinary stakeholders to apply evidence-based interventions to create, expand, or enhance urban green space in their communities. This paper presents a collaborative effort of experts representing the fields of landscape architecture, urban forestry, public health, social science, architecture, and climate change planning. This consensus review proposes a conceptual typology of practical interventions that integrate climate resilience and public health co-benefits in urban green space design and planning. We propose specific design interventions that are physical configurations of urban green space elements that address intentional combinations of key health and resilience benefits. Such intentions can apply to all materials of the built environment, though the focus in this paper is on landscape elements. The design interventions provide guidance to the diverse range of stakeholders necessary to champion urban forest and green space investment and design. The interventions also inform future research needs in green design, particularly in local community contexts.

### 1.1. The Intersection between Urban Green Space, Climate Change, and Human Health

The world’s climate systems are changing, and communities are exploring how to modify their environments to address current and future challenges [21]. Trees and vegetation can be used to provide regulating ecosystem services to help communities adapt to the negative impacts of climate change [3,22,23,24,25]. Urban forest research has quantified how trees can both mitigate greenhouse gas emissions [24,26] and be used to adapt to climate change consequences [27]. In addition, urban green spaces reduce vulnerabilities to predicted climate impacts through functions such as stormwater management [3], energy savings [4], providing shade and protecting thermal comfort [28,29,30], and reducing the urban heat island effect [31,32]. Local governments increasingly integrate climate change adaptation in decision-making about urban forests and other land-use planning processes [33]. Health authorities are also beginning to include climate projections in facilities planning [34].

Evidence also indicates that the global burden of disease is shifting to a higher prevalence of non-communicable diseases related to lifestyle [16,35,36]. Non-communicable, and often chronic diseases such as cancer, heart disease, mental illness, diabetes and obesity are affecting increasing numbers of people, particularly in the Global North and as populations age. Research is increasingly focusing on the role that urban green space plays in human health [37,38] as everyday living environments are social determinants that can mitigate or exacerbate disease, in the near and long term. Evidence indicates that urban green space has a positive effect on a range of health outcomes [39,40]. A positive health response to nearby nature may also reduce public health costs [41,42]. As human populations are increasingly concentrated in urban areas, there is a need to encourage greater opportunity for all members of society to have access to urban green spaces and gain health benefits. Yet, additional research about the specifics of nature and health is needed [37]. In addition, applied prescriptions for design and management of green spaces to maximize these benefits are still nascent; case studies of implementation and evaluative research are needed, and at multiple scales [43].

### 1.2. The Neighborhood and Block Scale

Scale is an important consideration for urban green space design and is a concern in many disciplines. The current knowledge on urban forest benefits specifies a range of scales, from tree, to street, to city-scale processes [3]. In a collaborative essay about the relationship between aesthetics and ecology, the authors argue that “the landscape scale of human experience … represents a strong entry point for transdisciplinary study” [44] (p. 960) of nature-society interactions. The neighborhood scale is an important and often missed opportunity to connect local residents with the interventions and practices of urban forestry and green space planning [44,45]. At this scale, design interventions and indicators connect with tangible and realizable outcomes that directly connect to people’s lives. As Opdam et al. argue, “local communities are where humans use landscapes to make a living and contribute to their quality of life, and where they adapt landscapes to create value from landscape services or prevent loss from external pressures such as climate change” [45] (p. 1441). Neighborhoods are a scale of importance to residents’ living quality [46].

Neighborhood scale thus represents an important social and perceptual landscape unit, but the concept and spatial extent of neighborhood is often not clearly defined. Steenberg et al. [47] used the term ‘neighborhood’ to define unique urban forest units based on established residential neighborhoods but using additional definitional elements beyond socio-political boundaries. Neighborhoods can be defined politically [48], socially [49], or geographically [50]. A neighborhood can range from a few blocks [51] to a larger area of the city encompassing many blocks [47]. Sheppard et al. [52] argue that the city block scale is an often neglected but promising level for community engagement and co-creation of climate change responses.

**Figure 1:**
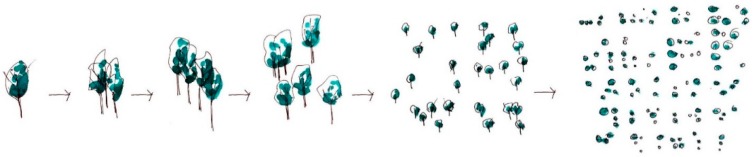
The scales of inquiry in urban green design interventions discussed in this paper range from the individual unit to the neighborhood scale. 1: Tree, 2: Grove, 3: Street, 4: Neighborhood park, 5: Block, 6: Experiential neighborhood.

For the purpose of this paper, we focus on what we term the experiential neighborhood. We define the experiential neighborhood as a cohesive unit of approximately 8–12 blocks, where a 10 min walk (800 meters) can take a person from one end of the area to the other [53,54]. Residents can realistically conceptualize this scale of neighborhood as a landscape unit, with a higher proportion of familiar social contacts and encounters, greater identity recognition, and strong place attachment [55]. This scale is common for campus environments such as hospitals and colleges. It has also been recognized as a suitable scale for mobility and tranquility planning in residential environments, as in the superblock concept of approximately 160,000 sq meters in Barcelona [56]. The neighborhood scale captures human green space experiences at shorter distances, allowing for consideration of accessibility, sightlines, aesthetics, vegetation layering, and quality of green space design. The experiential neighborhood scale is small enough to conceptually include the impact of individual trees, an important component of urban green spaces. As Duinker et al. argue, “[t]rees are special and unique in the plant community for many reasons … perhaps most profoundly because they predominate in contributing to the vertical dimension of the plant community on account of their height” [18] (p. 7380). Figure 1 below shows the range of scales that will be considered in the green design typology proposed in this paper, from the individual tree to the collective landscape of the experiential neighborhood.

## 2. Materials and Methods

Consensus review and engagement processes were used to prepare this paper, including extensive deliberative discussions and writing. Project partners contributed expertise and interests from diverse disciplines: climate change academics, urban forest academics, landscape architects, architects, facilities managers of local health authorities, municipal sustainability experts, and geospatial analysts. Input from the partners was obtained through iterative meetings, reviews and workshops over a period of twelve months.

An associated literature search included sources from multiple disciplines that may not typically intersect and focused on papers that supported measurement. Literature searches of electronic databases were carried out, and studies were limited to those published in English. Both review and empirical articles were included in search results, combining keywords: “green” or “green space (greenspace)” or “urban forest” or “green infrastructure”, coupled with “health” and/or “climate change”. The terms related to green spaces were used interchangeably, and health was interpreted as broadly as possible to include physical and mental health (e.g., well-being). Additional searches were carried out using reference lists and municipal resources to identify relevant public-sector reports (e.g., websites, practitioners). Approximately 30–40 papers were selected that described quantitative and qualitative relationships between green spaces, green infrastructure, urban forests, and human health.

The goal of the literature search was to identify a long list of potential indicators and metrics for relationships between green, health, and/or climate adaptation/mitigation (provided in the appendices). Metrics were field, sensor or human science measures that quantify a condition and/or change associated with an intervention. Metrics for population scale health benefits of urban green spaces included both qualitative and quantitative measures. The health metrics were interpreted into six indicator categories: physical access and general health, visual access, disease recovery, physical activity, mental health, and social support (Table A1). Similarly, Table A2 lists quantitative indicators relating to climate resilience benefits that are commonly cited in literature and frequently assessed using i-Tree software (USDA Forest Service; Syracuse, New York, NY, USA).

Our team of diverse intellectual and professional practice partners then engaged in extensive discussion of the applicability of the collected metrics across a range of potential neighborhood settings. When comparing the two tables, it became apparent that some neighborhood greening interventions aimed at either health or climate would improve outcomes for the other. Some metrics could be associated with more than one benefit or outcome of an intervention, even if the functional intentions were different. For example, measures of increased canopy cover can indicate improved urban heat island impacts, as well as increased available green space for local residents. Similarly, efforts to shade buildings for potential energy savings could create green views for hospital patients. Following processes of consensus, then synthesis, we generated a short list of conceptual design interventions that apply to smaller scale urban green space research and design (site, block, and neighborhood scale).

## 3. Results: A Conceptual Typology of Practical Green Design Interventions

We present a typology of novel interventions that provide critical functions through synthesis of the qualitative, tangible, and holistic aspects of neighborhood green spaces, with a focus on elements of urban forests and associated vegetation (Table 1). The intervention typology and illustrative diagrams that follow present various green space configurations and serve as a guidance system for urban greening to optimize human health and climate resilience co-benefits. The interventions achieve spatially explicit functions while enhancing quality of life and experience. Each of the eight interventions reflect:

- A primary health benefit and/or a climate resilience response identified from the literature review, and
- A physical vegetation configuration in relation to other structural elements and spatial conditions.

The proposed interventions could be applied to a variety of landscapes. However, we focus primarily on campus landscapes and the experiential neighborhood scale, featuring prominent mid-rise buildings with connective and/or peripheral open space elements, as might be found in health complexes, educational campuses, or mixed-use light industrial or multi-unit residential areas, with at least some public access to open space.

The eight design interventions and associated metrics (Table 1) comprise a potential experiential pathway of a site user as they traverse a neighborhood space. The closest “green experience” can occur without being outside: the nature elements only have to be visible from a window or door: *View from within*. As someone moves through their neighborhood, and as their activity periphery widens, they encounter other green spaces with varying characteristics of arrangement and structure. The final intervention, *Optimize green infrastructure*, encompasses all other interventions and operates at the largest scale. The interventions are therefore not mutually exclusive: for example, *Plant entrances*, *Create refuge*, and *Retain the mature* may all contribute to *View from within* and *Optimize green infrastructure*. Full descriptions and rationales for each intervention are provided in Section 3.1, Section 3.2, Section 3.3, Section 3.4, Section 3.5, Section 3.6 and Section 3.7 below.

The design interventions address the needs of a broad range of stakeholders, including site users and general members of the public; nearby residents; onsite staff and building occupants; facility managers and maintenance workers responsible for buildings and infrastructure; and urban planners. The design interventions should be particularly useful to policy and decision makers focused on health and climate issues, as the designs simultaneously address two comprehensive, relatively new and increasingly important community concerns.

The typology is a scalable toolkit to inform urban landscape design, using relatively simple quantitative and qualitative assessment metrics. Additionally, design detailing, including the planting pattern, species choices, and quantity of trees, will play an important role in determining resulting benefits. The eight design interventions provide guidance for designers, planners, and practitioners to choose evidence-based options for greening smaller scale urban areas.

### 3.1. View from Within

**Figure 2:**
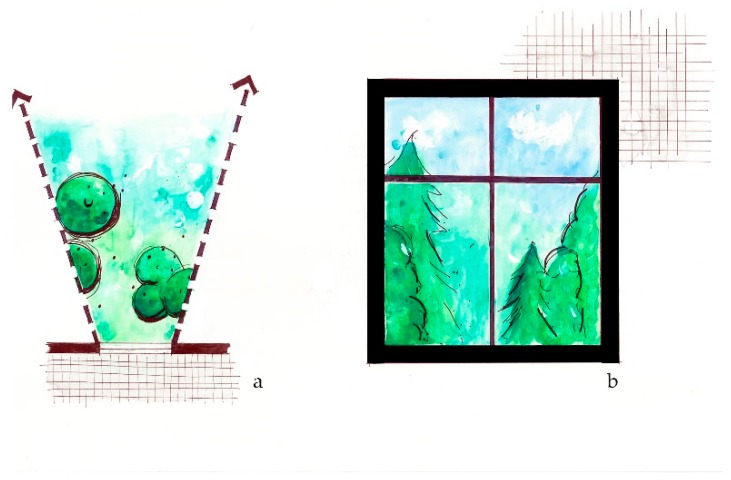
The *View from within* intervention considers green view opportunities for building occupants, such as trees, plants, water, or distant landforms, from the inside of a building. (**a**) Plan view sketch of window looking over landscape; (**b**) Sketch elevation of potential view from a window.

*View from within* refers to the ability to see a certain number of natural objects, such as trees, plants, water, or distant landforms, from the inside of a building. Evidence has shown that physical proximity to green space can promote physical and mental wellbeing. Evolutionary psychology theories, namely the “biophilia hypothesis”, suggest that humans also experience positive emotional responses from simply having views of nature. For example, when compared against other works of art, hospital patients may prefer looking at pictures and images of natural landscapes such as forests [57]. In a health care setting, viewing landscapes dominated by plants, flowers, trees, and other greenery can produce a significant restorative effect within a few minutes [58]. Studies have shown that even 40 s of green roof views, for example, can improve cognitive performance and boost attention span [59]. Visual access to green space and highly visible landscape features such as tall trees should therefore be included as a design intervention for health, particularly where outdoor exposure to green is not always possible, or is infrequent (Figure 2). This may be the case for hospital patients confined to the indoors, schoolchildren and students who spend most of their time in the classroom, and office workers.

Considering visibility as a design intervention also promotes the creation and management of green space in less traditional spaces and at varying levels. Green roofs, vertical gardens, and green walls can play an important role in improving access to visible green space, particularly in higher-density neighborhoods, where development occurs upwards rather than outwards, and in cities with increasingly high demand for physical space. Additionally, such biophilic building designs offer direct benefits for building efficiencies, by offering temperature moderation and energy savings. *View from within* can also incorporate distant views to natural spaces such as mountains and water bodies, and ensure the sightlines are preserved or enhanced. Seasonality should be considered when implementing the *View from within* intervention. Green views should be available year-round, and seasonal color could enhance the view at certain times of the year.

### 3.2. Plant Entrances

**Figure 3:**
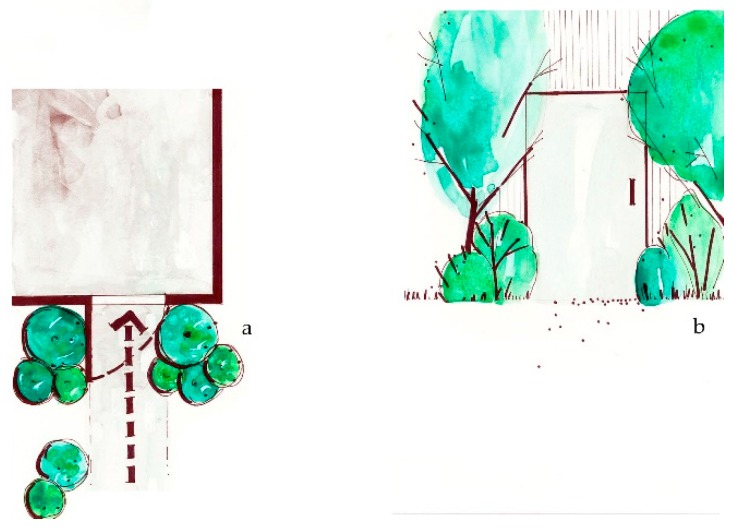
The *Plant entrances* intervention considers the amount of planted landscape at building or site entrances or exterior doorways, which may include trees or other vegetation. (**a**) Plan view of building entrance; (**b**) Sketch elevation of potential green entrance.

*Plant entrances* refers to the presence of green, which may include trees or other vegetation, at building or site entrances or exterior doorways. Having green elements in close proximity to site or building entrances serves multiple purposes (Figure 3). Depending on the location of the building entrance and vegetation, trees can provide shade, cooling effects, and subsequent energy savings. Building entrances are high-traffic areas as well as social spaces, drawing occupants to the outdoors as well as welcoming occupants inside. The presence of vegetation at building or site entrances ensures that all users are exposed to the associated green benefits as they enter and exit. In a study of landscapes surrounding social housing, Kuo et al. [60] found that residents spent more time outside if trees were closer to apartment buildings. This facilitated increased social interaction and supervision of children in otherwise isolating environments. In the case of high-rise buildings, vegetation near entrances also shortens the amount of time it would take for occupants to reach some level of green exposure.

The presence of green space at entrances is not a commonly used metric in green space or urban forest evaluation. When applied appropriately, however, smaller-scale interventions such as these are generally more feasible compared to larger scale interventions in terms of financial, legal, and other resource constraints [61,62]. Concerns about safety and security should be considered when designing plantings near entrances to ensure that the spaces created feel welcoming to all members of society.

### 3.3. Bring Nature Nearby

*Bring nature nearby* refers to the presence of green within close proximity of all neighborhood dwellers, regardless of demographic, cultural or socio-economic conditions. Example may include “pocket parks” and linear greenways. Exposure to green space, along with its associated benefits, has been shown to correlate with demographic and socio-economic conditions, often noted as disparities in availability of parks and trees in underserved communities [63]. Cities are increasingly recognizing the importance of green space accessibility, with organizations developing standards for nature provisioning [64]. A study in Europe found positive association between time spent in green space and mental health [65], and another found access to green spaces positively associated with mental health [66]. It is vital for urban forest managers to ensure that all community members have equal access opportunity to the physical and psychological benefits provided by urban trees.

**Figure 4:**
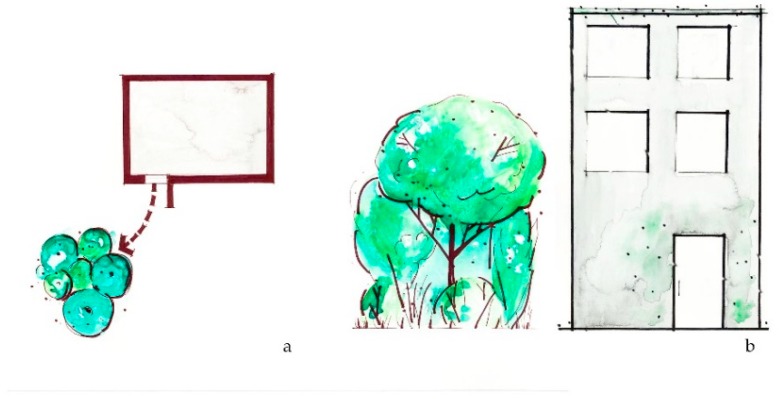
*Bring nature nearby* suggests providing close access to green for all member of society, including adding trees and plants within close proximity of everyone, regardless of demographic, cultural or socio-economic conditions. (**a**) Plan view of nearby nature; (**b**) Sketch elevation of green space near to a building.

Table 1 includes a novel metric which has not yet been tested: vertical distance to green space (Figure 4). Do urban dwellers who live twelve stories up experience fewer green benefits compared to those living on the ground floor, due to the time it takes to reach a certain amount of green? Do building users respond similarly to nature placed on multiple floors in biophilic buildings compared to ground plane landscapes? Larger cities are becoming increasingly dense, and residential high-rise buildings are prevalent in areas having higher housing demand due to restricted physical space and rising housing costs. It is therefore essential to develop and test green space indicators that account for changes in city demographics and urban planning trends.

### 3.4. Retain the Mature

**Figure 5:**
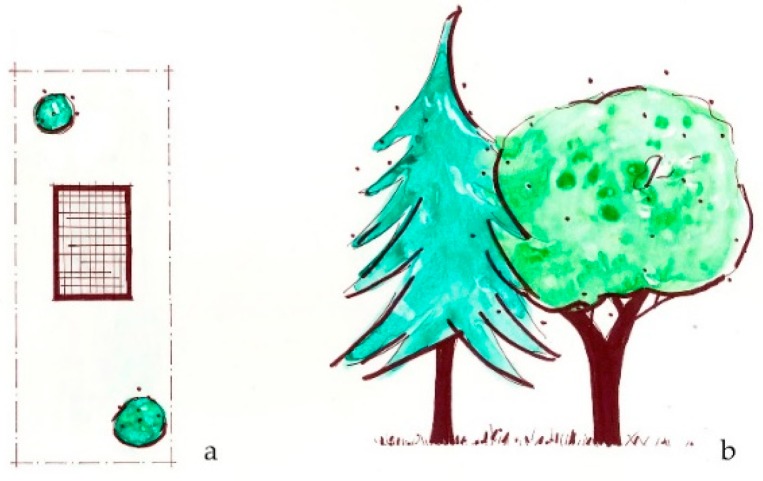
*Retain the mature* encourages retention of larger specimen trees as important components of the planted landscape, and that size and structure of trees are prioritized in green space planning. (**a**) Plan view of mature trees; (**b**) Elevation sketch of mature trees.

*Retain the mature* refers to paying attention to the size and structure of trees comprising a green space. Given the benefits provided by big trees, nature contact spaces could be designed around a “heritage” or “legacy” tree (Figure 5). Age diversity is important, particularly along city streets, to ensure that many trees are not removed simultaneously at the end of their lifespan. In the context of climate mitigation, one large tree sequesters and stores more carbon than a smaller one [24]. In terms of adaptation, a big, mature tree is also generally understood to provide more benefits compared to one of a smaller stature, such as more shade, greater energy savings, and air quality improvements [67].

Table 1 includes metrics for the *Retain the mature* intervention. Tree size [68], and structural diversity [69], presence of heritage trees [70], and larger trees [71] can be used to assess public perceptions of urban green space structure.

### 3.5. Generate Diversity

**Figure 6:**
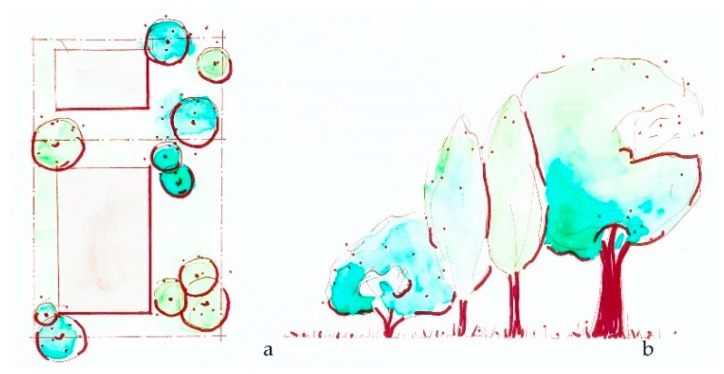
*Generate diversity* intervention suggests that a mix of plant species of trees and plants within a greenspace is aesthetically pleasing and will increase resilience to future changes in climate. (**a**) Plan view of diverse tree plantings; (**b**) Sketch elevation of diverse tree types.

*Generate diversity* refers to biological and structural and diversity in species of trees and plants comprising a green space. Species diversity (Figure 6) is crucial to urban forest functioning, resilience to pests and disease, and to the enhancement of green benefits. Research has shown that greater species richness is an important mitigator of environmental stressors, including tree pests and disease [72]. The presence of native tree species has been shown to promote the establishment of other native organisms, such as insects and birds, increasing the ecological integrity of urban forest ecosystems [5]. Many are widely used in assessing the quality of an urban green space, namely naturalness and ecological integrity (e.g., representation of native species), the number and representation of species present, Other qualitative indicators such as perceived safety [40] and green design aesthetics [73] relate to the diversity of planted landscapes.

### 3.6. Create Refuge

**Figure 7:**
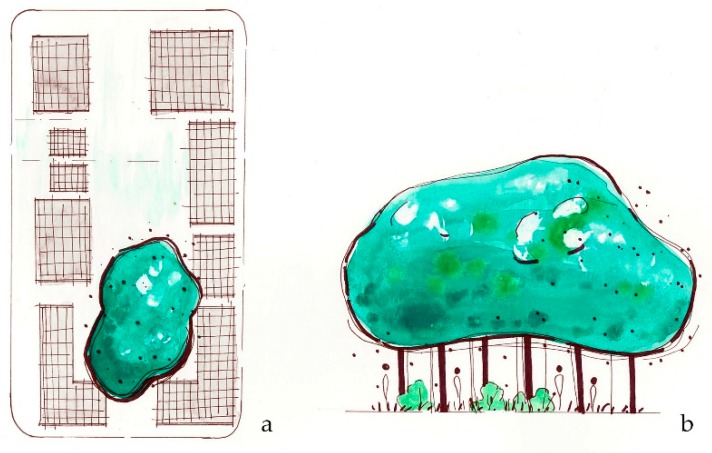
*Create refuge* suggests that shade from a large area of continuous canopy can provide shelter during a heat waveby providing “cool spots” where neighborhood dwellers can find protective temperatures. (**a**) Plan view of green refuge area; (**b**) sketch elevation of refuge area.

*Create refuge* refers to the presence of “cool spots”, where neighborhood dwellers can find protective temperatures during extreme heat events. An example would be a stand of trees with sufficient diversity in size and structure for shade and cooling due to evapotranspiration. Urban areas are increasingly subjected to the effects of climate change. Extreme heat events may compound the impact of the more general urban heat island effect, prompting municipal researchers and practitioners to recognize the importance of urban green in mitigating higher temperatures [27]. As illustrated in Figure 7, creating refuge allows urban dwellers to access public and green spaces in warmer temperatures, and can mitigate associated health risks [74].

Canopy cover percentage can provide some information on the estimated amount of shade that trees provide. At the site or block level, shade is an essential component of “cool” spots, particularly in relation to UV radiation exposure [75]. Reflecting increased concern of the public health impacts of urban heat events, studies and models provided knowledge about thermal comfort and perceived heat [76,77] including radiant temperatures [78], tree species effects [79], and tree planting configuration [80]. We include another novel metric, namely the number of people that a cool refuge can accommodate at once. The focus on people provides a tangible and accessible metric and can be calculated as a percentage based on the total population that might have access to a particular refuge or series of green spaces.

### 3.7. Connect Experiences

**Figure 8:**
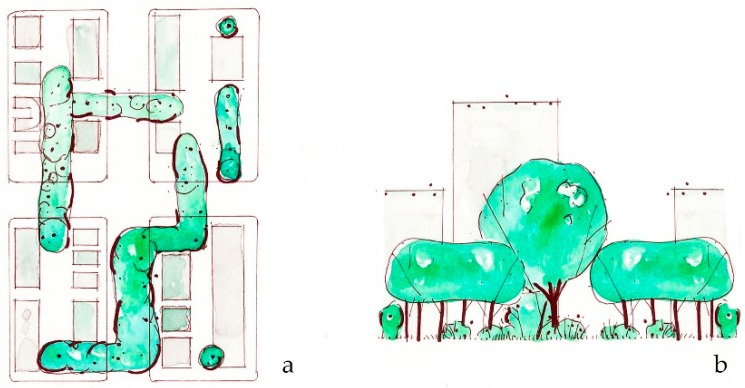
*Connect experiences* describes how having a continuous, pleasant walking environment can increase physical activity by providing greenery along streets or paths. (**a**) Plan view of connected green spaces; (**b**) Sketch elevation of connected greenery.

*Connect experiences* refers to continuous greenery at eye-level along a street or other transit paths, meant to encourage active transit and other forms of physical activity. Green space connectivity is an important consideration in urban wildlife management and biodiversity conservation and promotes species dispersal in fragmented landscapes [81]). Concepts such as “green corridors”, “parkways”, or “greenways” can be similarly applied to human movement, particularly in urban areas. Routes with sufficient trees, vegetation, and open space can serve as an escape from urban stresses like noise, traffic, and pollution [82]. Green corridors or roads can provide ready access to and between public open spaces, including green spaces (Figure 8). Evidence has also shown that the presence of greenery encourages physical activity; the presence of street-level green space may positively correlate with increased time spent walking [83]. People in dense urban environments are also more incentivized to walk to their destination when street trees are planted closer together [84]. Urban streets have the potential to provide a space for both transportation walking, to reach a destination, as well as recreational walking for pleasure, stress relief, and other health reasons [83].

We propose green space connectivity as a design intervention, particularly given its close relationship with accessibility to green benefits. Trees in particular arrangements (e.g., tree-lined paths) can create more accessible and aesthetically pleasing areas for pedestrian traffic; these ‘shadeways’ also create cooler walking and cycling routes during extreme heat events. While accessibility measures should ensure that all community members have equal opportunity for green exposure, connectivity design interventions are meant to layer this with additional physical activity and active transit encouragement.

### 3.8. Optimize Green Infrastructure

*Optimize green infrastructure* refers to sufficient canopy cover and other green infrastructure services to support a healthy and resilient living environment. In response to human health, having enough vegetation to filter the air can help mitigate air pollution, noise pollution, and visual stressors that can detract from wellbeing. From a climate change perspective, urban heat islands can be mitigated with sufficient tree canopy and vegetation to provide shade and evapotranspiration. Recent research suggests aiming for 40 percent canopy cover: a study focused on urban heat islands suggests that areas with canopy cover greater than 40 percent had significantly reduced daytime air temperatures [31], in another study, subjects reported increased stress reduction up to approximately 40 percent canopy cover [85].

**Figure 9:**
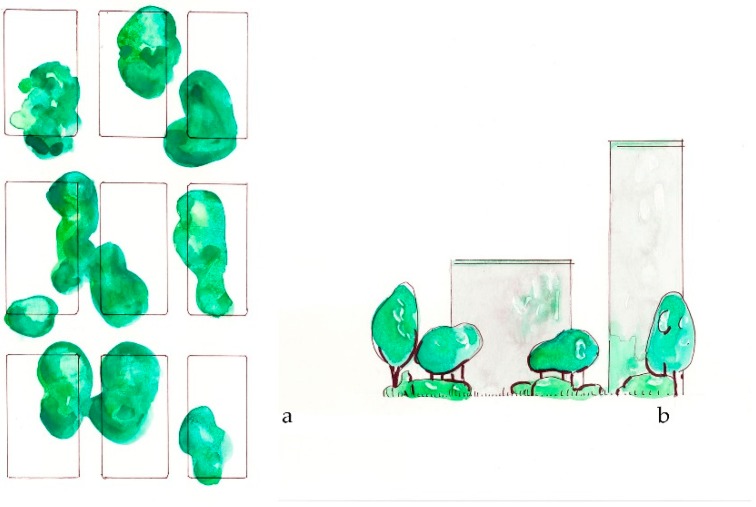
*Optimize green infrastructure* suggests that canopy cover, planting spaces, and pervious surfaces could be optimized within a neighborhood to maximize health and climate co-benefits. (**a**) Plan view of combined green spaces; (**b**) Sketch elevation of green spaces across blocks.

Canopy cover is commonly used to evaluate a city’s urban forest and is often used as a proxy for urban forest quantity [86]. Tree canopy cover is comprised of all trees making up the urban forest, as highlighted in Figure 9. Many cities set tree canopy targets to guide urban forest decision-making and management. Higher rates of tree canopy cover in a neighborhood is associated with higher potential for climate adaptation. For example, greater canopy cover would have greater heat island mitigation through increased shade and evapotranspiration [87,88]. It is also associated with human health benefits, such as better infant birth weight outcomes in neighborhoods with higher tree canopy cover [89,90].

While the recent studies cited above suggest a quantity to aim for, more research and context-specific exploration would be needed to create a specific target for a given neighborhood. Generally, those living in environments with more high-quality green space tend to report better physical and mental health outcomes [8,65]. More specifically, positive health outcomes may be due to pathways of reduced stress [91], increased social cohesion and interaction [92], and opportunities for exercise and physical activity [64,83]. Green exposure may include parks, natural areas, streets lined with mature trees, or any public place with sufficient green cover.

It should be noted that while green space provides important benefits, increasing green space within neighborhoods has been associated with negative impacts, such as gentrification [93]. Nonetheless, recent modeling studies emphasize the importance of measuring daily accessed greenery, rather than relying on top-down remote sensing (such as canopy cover), as the two measurements may not be equivalent in conveying a city resident’s experience [94,95]. While overall tree canopy and green space should be considered when planning or designing a community’s blocks, our typology suggests the need for future research that aligns landscape vegetation assessments with block-level experience.

## 4. Discussion

The interventions typology simplifies the myriad dimensions of green space experience and nature-based benefits into an easily understood, logical and memorable visual lexicon for use by practitioners from various disciplines. Our block-scale landscape interventions simultaneously address the importance of climate and health in green space design and management. While presented individually, the design interventions and associated co-benefits metrics function best when used in concert. For example, Norton et al. [27] suggest that having a diverse urban forest structure is important to ensure that heat does not get trapped under a continuous canopy of trees of the same species and size. This suggests consideration of how *Generate diversity* and *Create refuge* might interact. Similar situations can be presumed for additional combinations of interventions.

The feasibility of this typology across communities will probably depend on local and site-specific needs. Some indicators and metrics will be more relevant than others, and case studies can be used for further research and evaluation of health and/or climate change situations. Different priorities and different locations within urban areas will require a particular and context-specific suite of green space and urban tree planting responses [43,96]. For example, it has been shown that positive mental health impacts may depend on spatial scale and type of green space [91]. Additionally, in the health care context, an acute care center, where patients are not mobile, might prioritize visual access to nature. Visitors and staff would additionally benefit from some amount of physically accessible green space in this situation, potentially to reduce stress and burnout [97]. Conversely, in residential care context, accessible and equitable green space for social interaction and horticultural therapy might be prioritized [98]. It would also be important to examine variability in site user response, with sufficient regional and cultural representation, to elicit finer-scale information about impacts of actual interventions on people.

This review had some limitations. Due to project constraints and the early stage of this scholarship, it was not possible to include non-expert stakeholders such as open space users or health facility patients in the review (and co-design) process. However, information on public perceptions and social/psychological impacts was integrated wherever possible from relevant research literature, as well as practitioners’ experiences with their non-expert constituents. Future research should test the impact and appeal of these interventions for local residents and site users, as well as urban forest and facility decision makers.

Other research opportunities include operationalization, followed by testing and assessment of the typology. Further interdisciplinary critique and pilot testing is needed to refine the typology, including post-construction and occupancy evaluation of development on actual sites. Priorities for such research could include evaluating the applicability and effectiveness across different kinds of campus environments (e.g., health care facilities, institutional settings); assessing the usefulness of specific metrics for varying contexts; and, exploring the uptake of place-based stewardship for ongoing green space monitoring and maintenance.

The relationship of the typology to emerging urban green infrastructure policy, planning and management is another research opportunity. One possibility is the assessment of the degree of uptake and implementation of the green design interventions by professionals, decision makers, and community members. Various sustainability and resilience certifications could incorporate the typology, such as the SITES (The Sustainable Sites Initiative) or LEED-ND (Leadership in Energy and Environmental Design-Neighborhood Design).

A major challenge for any green intervention is management and maintenance. Municipalities often face financial and bureaucratic constraints for urban forest and green space funding, and depending on jurisdiction, dedicated and sustained funding mechanisms for urban greening are not always available. Assessing how local neighborhoods and communities adopt and adapt the intervention typology may indicate opportunities for community-based urban forest and green space stewardship. Place-based stewardship programs have been shown to benefit both the biophysical environment and involved community members through engagement and environmental education, and such programs have the potential to close the gap between what government and public institutions provide and what communities need [99,100]. These initiatives could also uncover the usefulness of specific metrics for specific social contexts, enabling communities to play an active role in identifying and implementing the most relevant metrics for their locale.

## 5. Conclusions

This paper addresses two major gaps in the urban greening literature and urban natural resources planning and design. The first is the need to consider green space co-benefits for improving both human health and climate resilience. The second is the use of the small neighborhood/campus scale as a critical experiential context for positive green space interactions and design interventions. We propose a typology of eight green design interventions that can be used to engage health professionals, urban forest practitioners and managers, and stakeholders in design, assessment, and decision-making for both climate and health co-benefits.

The typology that we have presented is based on published evidence and expert insight gained from an interdisciplinary consensus process that included a literature review and metrics extraction to develop urban green design interventions for human health and climate change resilience. We recognize that our list of metrics is not necessarily exhaustive, but rather a “jumping-off” point that synthesizes what is known to date. We expect the list to change as further research is carried out and more evidence emerges to inform additional interventions, particularly through direct application of the typology.

“Greener” strategies require local-level planning, governance, and implementation to respond to context-specific needs, emphasizing the importance of collaboration across government levels and sectors. The green design typology was a product of a co-creation process involving multiple stakeholders and disciplinary expertise, representing a variety of mostly professional experts. This typology can also be used as an opportunity to reach out to actors who may not have traditionally been involved in urban forest and green space design and management at the experiential neighborhood scale, such as health authorities, health care workers, sustainability practitioners, facilities managers and community members. This typology guides the implementation of smaller-scale greening initiatives, with the ultimate goal of effective public engagement and participation in developing appropriate and purposeful metrics for evaluating green space functions and success.

## Figures and Tables

**Table 1:** Conceptual typology of practical green design interventions and their associated climate and health co-benefits and metrics.

Design Intervention	Anticipated Climate and Health Co-Benefits	Green Conditions Metrics
1. *View from within*	Visual biophilic experiences Wildlife habitat and biodiversity Stormwater mitigation	% population who can see green on a daily basis from within buildings
2. *Plant entrances*	Social gathering space Orientation/navigation Shade provisioning/cooling Building energy savings (depending on aspect)	# trees/shrubs flanking a building entrance % vegetation cover around building/site entrance # buildings per block with ‘green’ entrances
3. *Bring nature nearby*	Social gathering space Shade provisioning/cooling Wildlife habitat provision and biodiversity Stormwater mitigation	Horizontal and vertical distance (or time) to reach closest green space Available green space per capita (green space density) % of population who see green on a daily basis Level of community ownership and decision-making power Diversity metric
4. *Retain the mature*	Air filtration Visual biophilic experiences Social gathering space Shade provisioning/cooling Stormwater mitigation Building energy savings Carbon storage and sequestration Wildlife habitat provision and biodiversity	Naturalness (# native species, canopy stratification) Species richness and evenness Size (e.g., DBH, height) diversity Perceived safety and condition Presence of heritage tree
5. *Generate diversity*	Visual biophilic experiences Wildlife habitat provision and biodiversity	Diversity index of tree species Diversity index of planted space types
6. *Create refuge*	Social gathering space for cohesion and enhanced social capital Shade provisioning Air filtration Wildlife habitat and biodiversity	# people who can experience cool refuge at once % canopy cover in a given site at high noon during periods of expected heat Level of “shelter” provided by vegetation (stand density) % population within 400 m of a cool refuge spot of X size.
7. *Connect the canopy*	Visual biophilic experiences Shade provisioning/cooling Wildlife habitat provision and biodiversity (e.g., ecological corridors) Stormwater mitigation	# active transportation (e.g., walking/biking) around green space Presence and # of physical barriers to green space (can also be the reverse, so the presence and # of paths leading to green space) Colorfulness, and arrangement (# tree-lined walks)
8. *Optimize green infrastructure*	Urban Heat Island mitigation Carbon storage and sequestration Stormwater mitigation Wildlife habitat provision and biodiversity	% cover Canopy volume Leaf area index (LAI) Area of green space

## Appendix A

**Table A1:** Health indicators.

Indicators	Metrics	References
1. Physical access and general health (e.g., exposure)	Noise level and exposure in green space Level of variation, naturalness, color Clear arrangement Shelter (open/closed) Walkability of green space Perceived safety and condition (e.g., maintenance, presence of tree pest) of green space Connectivity between and of green spaces to other trails, bike paths, etc. Tree size and urban forest structure Proximity to green space (horizontal) % green space Mean Normalized Difference Vegetation Index value within spatial area Size of green space Available green space per capita	[8,12,13,14,42,48,65,71,88,101,102,103,104,105,106]
Visual Access	% of population who can see green on daily basis	[58,59,107,108]
Disease and Recovery	Presence of indoor green infrastructure % self-reported disease incidence Length of recovery time during hospital stay % disease recurrence % walkouts (e.g., emergency patients who leave) # health complaints	[10,13,84,109]
Physical Activity	Presence and # of physical barriers to green space (e.g., major roads, presence of ramps for wheelchairs, etc.) # and proportion of tree-lined walks based on total area Perception of greenness and design aesthetics (e.g., “green qualities”) Proximity to green space (horizontal)	[13,15,54,101,102,106,110]
Mental Health	# of times people use green space Presence of water features in green space Presence of winding (non-linear) paths and trails Presence of green infrastructure at building entrance Perception of greenness and design aesthetics (e.g., Perceived Restorativeness Scale) Tree size and urban forest structure Proximity to green space (horizontal) % of green space	[7,10,41,65,98,101,111,112,113]
Social Support and Integration	Presence of infrastructure for social gatherings (e.g., seating) # encounters with other green space users # of people utilizing public and green spaces Average amount of time spent in green space Ownership and decision-making power around green space (e.g., community gardening) Proximity to green space Size of green space	[11,12,13,14,60,113,114]

## Appendix B

**Table A2:** Climate indicators.

Indicators	Metrics	References
Urban Heat Island	Canopy cover/volume Temperature below canopy	[30,31,32,77,90,115,116,117]
Greenhouse Gas storage/sequestration	GHG sequestration and storage Amount of biomass	[24,25,26,118]
Building energy	Energy conservation Percent of south/west building facades shaded	[4,23,25,51,76,118]
Stormwater control	Amount of pervious surface	[3,119]
Air quality improvement	Air quality	[25,91,118]
